# Influence of dietary habits on depression among patients with rheumatoid arthritis: A cross-sectional study using KURAMA cohort database

**DOI:** 10.1371/journal.pone.0255526

**Published:** 2021-08-05

**Authors:** Hiroto Minamino, Masao Katsushima, Motomu Hashimoto, Yoshihito Fujita, Mie Torii, Kaori Ikeda, Nozomi Isomura, Yasuo Oguri, Wataru Yamamoto, Ryu Watanabe, Kosaku Murakami, Koichi Murata, Kohei Nishitani, Masao Tanaka, Hiromu Ito, Miyabi Uda, Kazuko Nin, Hidenori Arai, Shuichi Matsuda, Akio Morinobu, Nobuya Inagaki

**Affiliations:** 1 Department of Diabetes, Endocrinology and Nutrition, Graduate School of Medicine, Kyoto University, Kyoto, Japan; 2 Japan Society for the Promotion of Science, Tokyo, Japan; 3 Department of Rheumatology and Clinical Immunology, Graduate School of Medicine, Kyoto University, Kyoto, Japan; 4 Department of Advanced Medicine for Rheumatic Diseases, Graduate School of Medicine, Kyoto University, Kyoto, Japan; 5 Department of Clinical Immunology, Graduate School of Medicine, Osaka City University, Osaka, Japan; 6 Department of Human Health Sciences, Graduate School of Medicine, Kyoto University, Kyoto, Japan; 7 Laboratory of Nutrition Chemistry, Division of Food Science and Biotechnology Graduate School of Agriculture, Kyoto University, Kyoto, Japan; 8 Department of Health Information Management, Kurashiki Sweet Hospital, Okayama, Japan; 9 Department of Orthopaedic Surgery, Graduate School of Medicine, Kyoto University, Kyoto, Japan; 10 Department of Orthopaedic Surgery, Kurashiki Central Hospital, Okayama, Japan; 11 National Center for Geriatrics and Gerontology, Aichi, Japan; Chiba Daigaku, JAPAN

## Abstract

**Objective:**

Although mental disorder is one of the most common comorbidities of rheumatoid arthritis (RA) and is known as a critical influence on RA remission rates, there is little knowledge regarding a possible therapeutic strategy for depression or anxiety in a RA population. Most recently, clinical evidence of dietary improvement for depression has emerged in a general population, but the relationship between dietary habits and mental disorder has not been investigated in RA. The purpose of this study is to elucidate clinical associations between mental disorder (depression/anxiety), dietary habits and disease activity/physical function in patients with RA.

**Methods:**

A cross-sectional study was performed with 267 female outpatients from the KURAMA database. Using the Hospital Anxiety and Depression Scale (HADS), we classified the participants into three groups by depression state, and their characteristics were compared. Using the 20-items on the self-reported food frequency questionnaire, we investigated the relationship between dietary habits and depression or anxiety, adopting a trend test and a multivariate standardized linear regression analysis for the HADS score of depression or that of anxiety as a dependent variable.

**Results:**

According to the classified stage of depression, current disease activity (DAS28-CRP: 28-Joint RA Disease Activity Score-C-reactive protein) and the health assessment questionnaire disability Index (HAQ-DI) were significantly increased. Trend analyses revealed that the depression score was inversely associated with the consumption of three food (fish, vegetables and fruit) out of twenty as was the anxiety score with only fish intake. Furthermore, multiple linear regression analysis revealed that the depression score was negatively associated with frequent fish intake (≥ 3 times per week) (Estimate -0.53, *p* = 0.033), HAQ-DI score within normal range (Estimate -0.88, *p* ≤ 0.001) and MTX use (Estimate -0.60, *p* ≤ 0.023). For the anxiety score, multivariate analysis showed similar but not significant associations with variables except for HAQ-DI score.

**Conclusions:**

In a RA population, both depression and anxiety had a significant and negative association with HAQ-DI score, and depression rather than anxiety had negative association with frequent fish intake. Modification of dietary habits such as increased fish consumption may have a beneficial effect on the depression state in RA patients.

## Introduction

Rheumatoid arthritis (RA) is a chronic inflammatory disorder that causes joint destruction and physical disability. RA patients have increased risk for several comorbidities compared to the general population [1]; accumulated comorbidities worsen treatment response, mortality and quality of life [2, 3]. Although new RA therapeutics such as biological Disease Modifying Anti-Rheumatic Drugs (b-DMARDs) and Janus kinase (JAK) inhibitors can successfully improve joint inflammation [4], they are insufficient to reduce extra-articular manifestations including cardiovascular disease, sarcopenia and mental health disorders. Thus, comprehensive management of RA-related comorbidities remains a considerable unmet clinical need in the era of biologics.

Mental disorder is one of the most common comorbidities of RA, and the prevalence of depression and anxiety is approximately two times greater than that in the general population [5, 6]. Depression and anxiety have a critical influence on RA remission rates, as they worsen adherence to medication, treatment response, clinical symptoms (e.g., tender joints and fatigue) and functional status [7–9]. Mental disorder is known as risk for the emergence of RA [10], and is also closely interconnected by crosstalk between neurocircuits and inflammation [11], which are implicated in the pathophysiology of depression [12]. Psychological stress can activate inflammatory responses via several immunomodulatory pathways [13], and elevated inflammatory markers are clinically associated with resistance to conventional antidepressant therapy [14]. Although small-scale studies suggest that some kinds of DMARDs might improve mental health in RA patients [15] and that infliximab could attenuate treatment-resistance depression in medically healthy adults [16], there is little evidence regarding a possible therapeutic strategy for RA patients.

Recently, clinical evidence of dietary improvement as a lifestyle intervention for mental disorder has emerged in the general population. As shown in previous studies including meta-analyses and randomized control trials (RCTs) [17–19], fish, fruits and vegetables can ameliorate depression. These foods contain a wide-variety of nutrients beneficial for depression (e.g., calcium, magnesium, iron, vitamin C and folic acid) [19] and especially n-3 poly-unsaturated fatty acids (PUFAs) as demonstrated in prospective studies on depression and anxiety [20–22]. In addition, since many of these foods and nutrients are well known for their anti-inflammatory effects in various diseases including RA [23], they might be expected to bring benefits both on mental health and RA disease activity. However, clinical associations between dietary habits and mental disorders have not been analyzed in an RA population.

To elucidate the relationships among the depression state and dietary habits as well as the anxiety state and dietary habits in RA patients, we performed a cross-sectional study using the Kyoto University Rheumatoid Arthritis Management Alliance cohort (KURAMA) database.

## Materials and methods

### Participants and study settings

We established the KURAMA (Kyoto University Rheumatoid Arthritis Management Alliance) database in 2011 for the storage of clinical data and specimens obtained from RA patients. The frequency of visits for participants was at least once every three months. Each one rheumatologist per patient assessed and recorded RA disease activity including 28 tender and swollen joint sites in a template for electronic charts on every visit, and the obtained data were integrated with blood tests and other clinical information. We recruited female RA outpatients from the KURAMA cohort database [24]. Participants were enrolled from May 2014 to December 2014 who visited the Kyoto University Hospital, were over 18 years old, and met the diagnostic criteria of the ACR/EULRA RA classification [25]. Of a total of 365 outpatients included during the study period, those with incomplete responses to the food intake questionnaire and HADS questionnaire (n = 79, 21.6%) and with loss of data of current disease activity in 2014 (n = 19, 5.2%) were excluded. A cross-sectional study was performed with the remaining 267 RA patients (73.1%) to elucidate the association among dietary habits, RA disease state, and depression or anxiety state.

### Ethics

This study complied with the principles of the Declaration of Helsinki and its procedures, and protocols were approved by the Medical Ethics Committee of Kyoto University Graduate School and Faculty of Medicine (Approval number: E1308). Written informed consent was obtained from all subjects.

### Data collection

We obtained the information about dietary habits and HADS by a self-reported questionnaire. In most cases, the questionnaire and clinical data were obtained on the same day. If patients forgot to complete the questionnaire, they were asked to answer it at the next visit within three months.

### Evaluation of dietary habits

We obtained dietary records of RA outpatients using self-reported food questionnaire as previously reported [26, 27]. Participants recalled the frequency of food intake in the last year, and filled out a questionnaire form regarding how often they had eaten food and drink items on an eight-point category scale ranging from 1 = “< 1 time/month”, 2 = “1–3 times/month”, 3 = “1–2 times/week”, 4 = “3–4 times/week”, 5 = “5–6 times/week”, 6 = “1 time/day”, 7 = “2 times/day”, 8 = “3 times/day”. The following 20 items of foods and beverages were surveyed: (1–3) staple food (bread, noodles, or rice) for breakfast (1), lunch (2), and dinner (3), (4) meat, (5) fish, (6) *tofu* (soybean curd), (7) eggs, (8) milk, (9) vegetables, (10) fruits, (11) deep-fried foods, (12) cakes, (13) juice or isotonic drinks, (14) junk foods, (15) sweets like candies and chocolates, (16) frozen foods, (17) pickles, (18) processed food (ham, sausage or *kamaboko* (boiled fish paste)), (19) *miso* soup (fermented soybean paste) and (20) alcohol. For staple foods, we asked participants about breakfast, lunch and dinner separately. For other food and beverage, we collected the frequency of dietary habits throughout all meals of the day.

### Estimation of depressive and anxiety state

We assessed patients’ depression and anxiety state using the Hospital Anxiety and Depression Scale (HADS), which is widely used to evaluate emotional health of outpatients [28, 29]. HADS consisted of a 7-items questionnaire for depression or anxiety state and is scored on a 4-point Likert scale (range 0–3), with total scores ranging 0–21. For both anxiety and depression state, the following commonly used criteria were adopted to classify the groups: Scores between 0 and 7 = ‘no’, 8 to 10 = ‘possible’, and 11 to 21 = ‘probable’. The favorable internal consistency (> 0.85) for HADS subscale was established by several reports [29, 30].

### Estimation of clinical parameters

We evaluated RA disease activity and physical disability using a 28-Joint RA Disease Activity Score-C-reactive protein (DAS28-CRP) [31], the patient overall disease-Visual Analogue Scale (patient overall disease-VAS) [32], Steinbrocker’s stage and class, and the health assessment questionnaire disability Index (HAQ-DI) [33]. The clinical remission of current disease activity and patients without disability defined using DAS28-CRP and HAQ-DI as follows: DAS28-CRP < 2.6 and HAQ-DI ≤ 0.5 [31]. The data on current RA therapeutics including methotrexate (MTX), prednisolone (PSL) and biological agent were obtained from the KURAMA database. Other epidemiologic information including age, duration of RA disease, body mass index (BMI) were also collected from the KURAMA database.

### Statistical analysis

Continuous variables are expressed as the mean ± standard deviation (SD) or median (interquartile range: IQR), and categorical variables are expressed as numbers (%). For comparison of participant characteristics according to depression status, a Steel-Dwass test or a Fisher’s exact test was conducted for continuous variables and for categorical variables, respectively.

To investigate association between dietary habits and depression in RA patients, we first performed a Jonckheere-Terpstra trend test as a univariate analysis. We also adopted the same analysis for exploring the relationship between dietary habits and anxiety. After detecting significant variables in the frequency of food intake, we then opted for a multiple standardized linear regression analysis with each depression and anxiety score (HADS) as a dependent variable. In this multiple regression analysis, we adopted the following clinically relevant factors as simultaneous independent variables: disease duration (continuous variable), RA therapeutics (methotrexate, prednisolone and biological agents, 0: no, 1: yes), DAS28-CRP (0: < 2.6, 1: ≥2.6) and HAQ-DI (0: >0.5, 1: ≤ 0.5). We constructed the following three models: patients without disability (model 1), DAS28-CRP remission (model 2) or both variables (model 3) as covariates. As for the factors of dietary habits, because their distribution differs greatly among foods, we redistributed intake frequency into the following binary variables based on the median: fish (0: low frequency (≤ 2 times/weeks), 1: high frequency (≥ 3 times/week)), vegetable and fruits (0: low frequency (≤ 6 times/week), 1: high frequency (≥ 1 time/day)). Statistical analysis was performed by the use of JMP 15.2.0 (SAS Institute Inc., Cary, NC, USA) and SPSS Statistics 26 software (IBM, Armonk, NY, USA).

## Results

### Characteristics of participants

Baseline demographics are shown in Table 1. A total of 267 female patients with RA were subjected to the following analyses. The mean (± SD) age was 60.7 (± 12.8) years and the disease duration of RA was 13.4 (± 12.6) years. The following therapeutics were used: methotrexate in 74.2%, prednisolone in 28.1%, and biological agent in 46.1%. In the context of these treatments, the mean (± SD) DAS28-CRP was 1.87 (± 0.80) and the majority of the participants was under remission of disease activity (DAS28-CRP < 2.6, 80.1%). The proportion of patients without disability was 55.3% (HAQ-DI ≤ 0.5). As for the mental health of participants, according to HADS depression score, 12.0% were categorized as ‘probable depression’ (score ≥11), 15.4% as ‘possible depression’ (score 8 ~ 10)’ and 72.7% as ‘no depression’ (score ≤ 8). As for the anxiety state of participants, 8.6% were categorized as ‘probable anxiety’ (score ≥11), 9.0% as ‘possible anxiety’ (score 8 ~ 10) and 82.4% as ‘no anxiety’ (score ≤ 8) according to HADS anxiety score.

**Table 1:** Baseline characteristics of study population.

			RA patients				
Items			(*N* = 267)	Items			
Age, years			60.7 ± 12.8	Depression and anxiety			
Body mass index, kg/m2			21.9 ± 3.5		Depression score (HADS-D)		5.58 ± 3.74
Laboratory data						Score 0–7, *n* (%)	194 (72.7)
	Hemoglobin, g/dL		12.4 ± 1.4			Score 8–10, *n* (%)	41 (15.4)
	Albumin, g/dL		3.91 ± 0.33			Score 11-, *n* (%)	32 (12.0)
	CRP, mg/dL median (IQR)		0.1 (0–0.2)		Anxiety score (HADS-A)		4.66 ± 3.78
RA-related parameters						Score 0–7, *n* (%)	220 (82.4)
	Duration, years		13.4 ± 12.6			Score 8–10, *n* (%)	24 (9.0)
	DAS28-CRP		1.87 ± 0.80			Score 11-, *n* (%)	23 (8.6)
	DAS28-CRP remission, *n* (%)		214 (80.1)	Dietary habits ***			
	HAQ-DI score, median (IQR)		0.38 (0–1)		Fish dishes		3 (3–4)
	Patients without disability*, *n* (%)		139 (55.3)		Meat dishes		4 (3–4)
	Patient overall disease-VAS		27.7 ± 24.0		Egg dishes		4 (3–5)
	Stage**		2.73 ± 1.18		Vegetable dishes		6 (6–7)
		Stage I	55 (20.6)		Fruits		6 (4–6)
		Stage II	66 (24.7)		Milk		6 (3–6)
		Stage III	43 (16.0)				
		Stage IV	103 (38.6)				
RA therapeutics							
	Methotrexate use, *n* (%)		198 (74.2)				
	Prednisolone use, *n* (%)		75 (28.1)				
	Biological agent use, *n* (%)		123 (46.1)				

Data are presented as the mean (± standard deviation: SD) or mean (interquartile range: IQR) for continuous variables, and as numbers (%) for categorial variables. The remission of DAS28-CRP is defined as DAS28-CRP < 2.6.

* The patients without disability is defined as HAQ-DI ≤ 0.5.

* Steinbrocker’s classification.

*** Food intake frequency was divided as follows: 0 = seldom, 1 = < 1 time/month, 2 = 1–3 times/month, 3 = 1–2 times/week, 4 = 3–4 times/week, 5 = 5–6 times/week, 6 = 1 time/day, 7 = 2 times/day, 8 = 3 times/day.

Abbreviations: *RA* rheumatoid arthritis, *CRP* C-reactive protein, *DAS28-CRP* 28-joint Disease Activity Score using C-reactive protein, *HAQ* health assessment questionnaire, *VAS* visual analogue scale, *HADS* hospital anxiety and depression scale.

We also conducted a comparative evaluation of the patients included in the analysis and those excluded. As a result, we found that the exclusion group was older, and had a lower use of methotrexate and a higher use of biological agents than the inclusion group (S1 Table).

### Comparison of RA-related factors according to depression state

To ascertain participant RA characteristics according depression state, we separated the patients into three groups (No/Possible/Probable depression) and compared the RA-related factors (Table 2). As the stage of depression increased, the degree of current disease activity DAS28-CRP, patient overall disease-VAS was significantly increased, whereas age, disease duration and laboratory data including hemoglobin, albumin and CRP were unchanged. As for the HAQ-DI score, its score increased with the increase in depression score. Regarding therapeutic drugs, although there was no statistical difference, the use of methotrexate tended to decrease with the degree of depression, while the use of prednisolone tended to increase.

**Table 2:** Participants characteristics according to the depression state.

Depression state	No	Possible	Probable	*P* value *
HADS score	0 ~ 7	8 ~ 10	≥11	
(N = 267)	*n* = 194 (72.7%)	*n* = 41 (15.4%)	*n* = 32 (12.0%)	
Age, year	60.4 ± 13.6	63.0 ± 10.4	59.5 ± 10.7	0.42
Body mass index, kg/m2	21.9 ± 3.5	22.2 ± 3.8	21.4 ± 3.1	0.63
Laboratory data				
Hemoglobin, g/dL	12.5 ± 1.3	12.2 ± 1.7	12.2 ± 1.3	0.24
Albumin, g/dL	3.93 ± 0.30	3.85 ± 0.42	3.82 ± 0.33	0.086
CRP, mg/dL, median (IQR)	0.1 (0–0.2)	0.1 (0–0.3)	0.1 (0–0.2)	0.71
RA disease characteristics				
Disease duration, year	12.8 ± 12.3	14.2 ± 13.4	16.0 ± 13.8	0.39
DAS28-CRP	1.80 ± 0.76	1.89 ± 0.76	2.28 ± 0.95	**0.0065**
HAQ-DI, median (IQR)	0.38 (0–0.88)	0.63 (0.13–1.25)	0.88 (0.5–1.5)	**< 0.0001**
Patient overall disease-VAS	24.5 ± 21.9	30.4 ± 26.1	43.5 ± 27.3	**< 0.0001**
Stage				
Stage I vs. II, III, IV, *n* (%)	42 (21.7)	7 (17.1)	6 (18.8)	0.77
Stage I, II vs. III, IV, *n* (%)	90 (46.4)	17 (41.5)	14 (43.8)	0.83
Stage I, II, III vs. IV, *n* (%)	115 (59.3)	28 (68.3)	21 (65.6)	0.48
Current therapeutic agent				
MTX use, *n* (%)	155 (77.3)	28 (68.3)	20 (62.5)	0.14
MTX dose, mg	7.31 ± 2.87	7.57 ± 2.85	8.70 ± 2.85	0.12
Biological agent use, *n* (%)	91 (46.9)	16 (39.0)	16 (50.0)	0.59
Prednisolone use, *n* (%)	47 (24.2)	16 (39.0)	12 (37.5)	0.072
Prednisolone dose, mg	4.76 ± 4.49	4.22 ± 1.91	4.17 ± 2.04	0.83

Participants are divided into the following three groups for comparison: of characteristics: No (HADS score 0 ~ 7), Possible (8 ~ 10), and Probable (≥11). Data are expressed as the mean (± standard deviation: SD) or mean (interquartile range: IQR) for continuous variables, and as numbers (%) for categorial variables.

Abbreviations: *HADS* hospital anxiety and depression scale, *RA* rheumatoid arthritis *CRP* C-reactive protein, *DAS28-CRP* 28-joint Disease Activity Score using C-reactive protein, *HAQ* health assessment questionnaire, *VAS* visual analogue scale.

### Specific dietary habits including increased fish intake are significantly associated with depression state in RA patients

**Fig 1:**
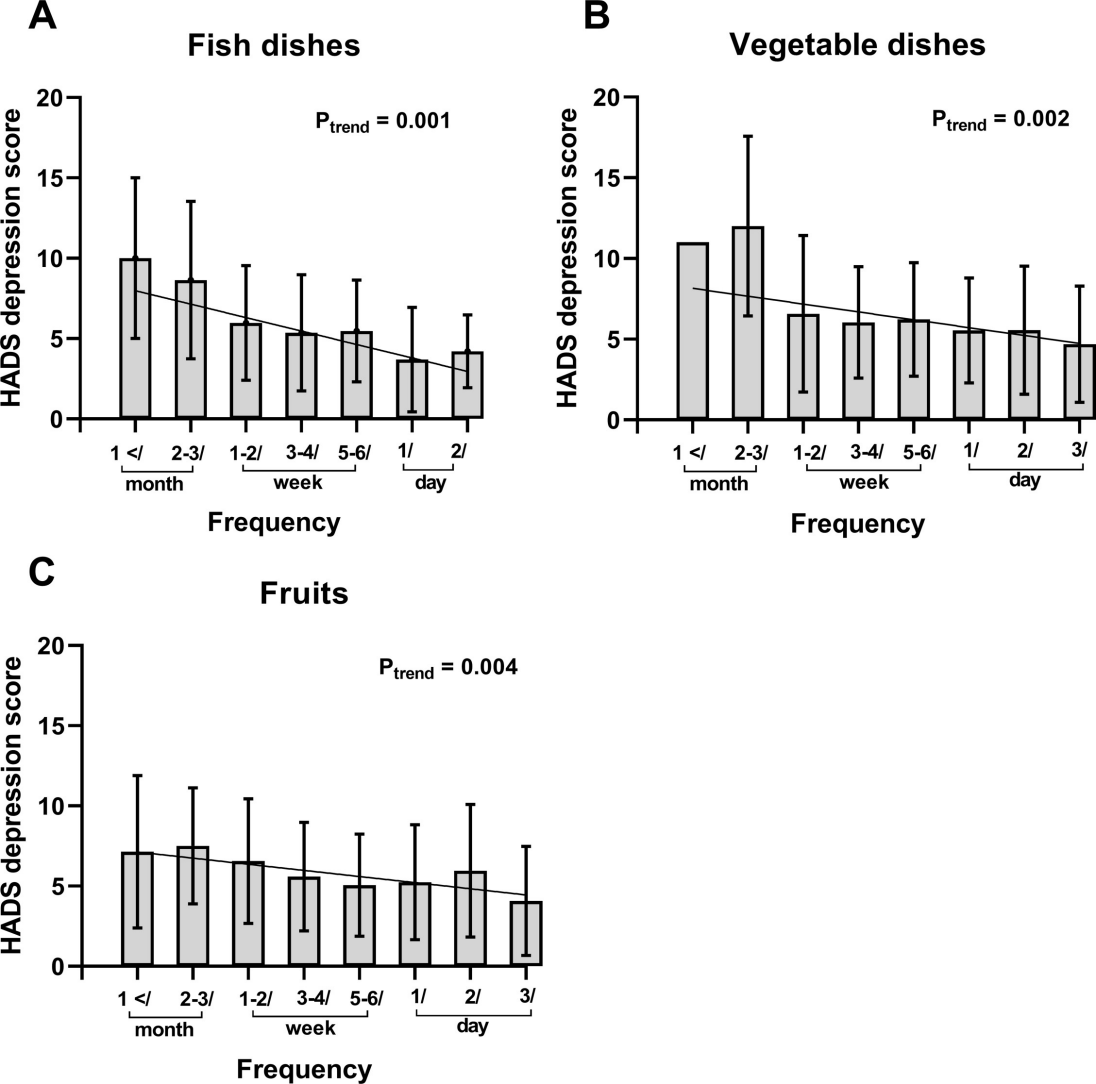
Trends of depression score by intake frequency. The vertical axis represents HADS depression score and the horizontal axis represents intake frequency of each category. 3 of 20 groups are negatively associated with HADS depression score; fish (A), vegetable (B), and fruits (C). P_trend_ values are calculated by a Jonckheere-Terpstra trend test. Abbreviations: *HADS* hospital anxiety and depression scale.

We next conducted the following analyses regarding the association between depression and food intake patterns. Trend analysis revealed that 3 out of 20 items taken at higher food intake frequency were negatively associated with the HADS depression score: fish (Fig 1A), vegetables (Fig 1B) and fruits (Fig 1C). Next, to identify whether these three dietary factors independently contributed to the depression state, we opted for a multiple standardized linear regression analysis with depression score (HADS) as a dependent variable. As a consequence, we found that in dietary habits, frequent fish intake (≥ 3 times/week) was inversely and significantly associated with depression score in the model that used RA-related factors and the patients’ status of disability (Table 3 left) (Estimate -0.53, *p* = 0.032). In other models using the remission of DAS28–CRP (Table 3 middle) or both variables (Table 3 right) as covariates, the same relationships were confirmed. (Estimate -0.55, *p* = 0.023 in Table 3 middle, Estimate -0.53, *p* = 0.033 in Table 3 right). The use of methotrexate and the presence of patients without disability were also significantly and negatively associated with depression score in all models. These results raise the possibility that frequent fish intake has a beneficial effect on the depression state of RA patients.

**Table 3:** Multivariate analyses for independent factors associated with depression scores including factors of dietary habits.

Dependent variables	Depression score	model 1		model 2		model 3	
Independent variables		Estimates	*p-*value	Estimates	*p-*value	Estimates	*p-*value
Dietary habits	Fish dishes	-0.53	0.032	-0.55	0.023	-0.53	0.033
	Vegetable dishes	-0.30	0.33	-0.28	0.36	-0.30	0.34
	Fruits	-0.13	0.61	-0.19	0.48	-0.12	0.65
Current disease activity	DAS28-CRP remission			-0.70	0.016	-0.26	0.41
Physical function	Patients without disability	-0.97	< 0.0001			-0.88	0.00080
	Prednisolone (+)	0.032	0.90	0.11	0.67	0.0050	0.99
	Biological agents (+)	-0.13	0.58	-0.016	0.94	-0.098	0.68
	Methotrexate (+)	-0.60	0.023	-0.65	0.013	-0.60	0.023
	Duration (10 years)	0.016	0.94	0.016	0.40	0.011	0.96

Results of multiple regression analysis with dietary habits and RA-related factors. Model was adjusted for RA duration, dietary habits (intake frequency of fish, vegetables and fruits), RA therapeutics (use of prednisolone, biologics and methotrexate) and the patients’ status of disability (model 1) or DAS28-CRP remission (model 2) or both remission scores (model 3).

Binary variables were constructed as follows: DAS28-CRP (0: < 2.6, 1: ≥2.6) and Patients without disability HAQ-DI (0: >0.5, 1: ≤ 0.5), fish (0: low frequency (≤ 2 times/weeks), 1: high frequency (≥3 times/week), vegetable and fruits (0: low frequency (≤ 6 times/week), 1: high frequency (≥1 time/day).

Abbreviations: *HADS* hospital anxiety and depression scale, *DAS28-CRP* 28-joint Disease Activity Score using C-reactive protein, *HAQ* health assessment questionnaire, *RA* rheumatoid arthritis.

### Dietary habits were not associated with anxiety status in RA patients

To clarify whether dietary habits are associated not only with depression but also anxiety, we next conducted the same analyses as we did for the depression state. Regarding anxiety state, trend analysis revealed that 1 of 20 items of higher food intake frequency were negatively associated with the HADS anxiety score: fish (S1 Fig). Using this significant dietary habit, we then performed a multiple linear regression analysis with anxiety score as a dependent variable. As a result, similar to the depression score, the presence of patients without disability was significantly and inversely associated with the anxiety score (S2 Table). However, unlike in the analysis of depression, no dietary factors were associated with anxiety score in either model (Estimate -0.25, *p* = 0.31)

## Discussion

The present study is the first to reveal a significant association between depression state and specific dietary patterns in a RA population. In this study, the prevalence of depression (12.0% as ‘probable depression’, and 15.4% as ‘possible depression’) was higher than that in the general population [34]. In addition, RA disease indicators (HAQ-DI and DAS28-CRP) were significantly higher in patients with increased HADS depression score, a commonly used scale for depression state. These results accord with previous findings (Cabrera-Marroquín et al.) that depression closely interacts with RA inflammation and has a pivotal influence on disease outcomes [35, 36]. In univariate trend analyses, the HADS depression score was inversely associated with intake frequency of fish, fruits and vegetables, and the HADS anxiety score was only with fish. Furthermore, in multivariate analyses, the HADS depression score was negatively correlated with frequent fish intake as well as with patient’s function and MTX use. These results accord with previous reports that depression reduces the RA remission rate [26, 36] and that MTX therapy might bring benefits on mental disorder as well as disease activity and HAQ [15]. Multivariate analysis for anxiety score showed similar but not significant associations with variables except for the patients’ status of disability defined by HAQ-DI score, which might be attributed to the HAQ status as a stronger covariate compared to other ones [37].

Accumulating evidence in the general population associates specific dietary habits and nutrients in the development or prevention of mental disorders. A meta-analysis confirmed that higher intakes of fish, whole grains, fruits and vegetables are associated with a reduced likelihood of depression and anxiety [38, 39], whereas dietary patterns including more processed food, sugary products and saturated fats may increase depression risk [40, 41]. In addition, recent RCTs have also shown that such healthy diets improve clinical levels of depression both in young and middle-aged adults [18, 42, 43]. Furthermore, fish-derived n-3 PUFA intake and n-6: n-3 ratio of plasma PUFAs are inversely associated with depression and anxiety [21, 22], prospective cohort studies in Japan have shown that higher intake of fish or its n-3 PUFAs are significantly associated with reduced risk for depression during the follow-up periods [20, 21]. Based on these findings and the results of the present study, specific dietary patterns may have beneficial effects on mental distress in RA patients, although the longitudinal influence of dietary habits are almost unknown and should be investigated.

**Fig 2:**
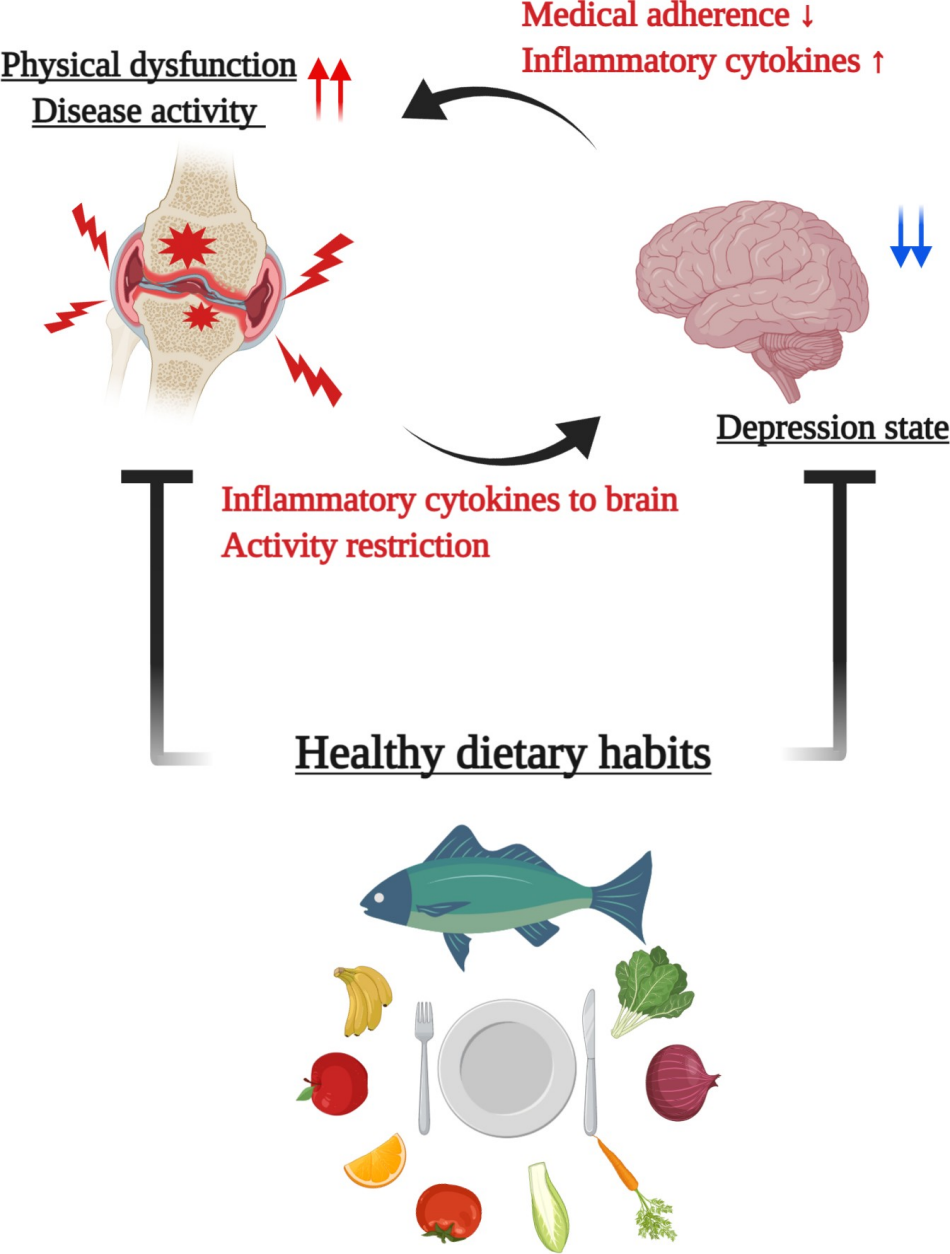
A model of the beneficial effect of healthy dietary habit on depression state in RA patients. Depression state and disease activity/physical disability in RA could be mutually influential as follows: depression affects decreased adherence to adequate treatment and is thought to promote inflammatory response via several immunomodulatory pathways, resulting in exacerbation of RA, which influences psychological distress because of increased pain and activity restriction and because of direct inflammation to brain. Healthy dietary habit including fish, vegetable and fruit intake may have a positive impact on both depression and disease activity and may be a potential therapeutic target to prevent exacerbation of the vicious cycle mentioned above.

Recently, the mutual interplay between systemic inflammation and depression has been spotlighted as a proposed cycle model of the exacerbation of RA [11]. Increased peripheral pro-inflammatory cytokines such as TNFα and IL-6 can affect blood-brain barrier permeability [44, 45], and are implicated in the pathophysiology of depression [12, 46]. Indeed, some genetic epidemiological studies have shown significant associations between the severity of depression and polymorphisms in inflammatory cytokine genes encoding IL-1β, IL-6, TNF and CRP [47, 48]. Psychological stress, in turn, is thought to promote the inflammatory response via several immunomodulatory pathways [12, 49, 50], and can also exacerbate clinical symptoms and impair medical adherence, treatment response, and physical activity [8, 9, 51]. In addition, a large-scale cohort study has shown that exposure to a stress-related disorder is a risk factor for subsequent autoimmune disease [52]. Thus, impeding this interconnection might be a possible candidate therapeutic target for depression in patients under chronic inflammatory condition; the current study provides clinical evidence that a healthy diet could be effective in the mental health portion of this cycle in RA patients. Furthermore, some of the beneficial diets for depression such as fish rich in n-3 PUFAs, fruits and vegetables are also well-known for their anti-inflammatory effects against RA disease activity, as shown in clinical reports including a previous one from our group [27, 53, 54].Taken together, dietary improvement would seem to be a fruitful co-adjuvant therapy for RA patients with mental disorders (Fig 2). Further research is required to see if dietary interventions could be a significant therapeutic option for mental health in RA patients, who are about twice as likely as the general population to develop depression.

The strong point of the present study is that we have combined the HADS score with a food frequency questionnaire (FFQ) for evaluation of both the depression state and dietary habits in a large-scale population study. The HADS score is well-known as a simplified and valid instrument for assessing depression and anxiety, and is often applied to medical patients including those with RA [29]. FFQ is also a widely used method for quantitative evaluation of eating habits in RA patients as well as in the general population [26, 27]. Our combination of reliable self-reporting forms is a versatile platform for evaluating dietary habits and depression/anxiety state in various study populations, and can potentially provide new insights into clinical applications of nutrition therapy.

There are several notable limitations in the present study. The cross-sectional study does not imply causation and further longitudinal research is needed. Only female participants were enrolled, although female sex is highly dominant both in patients with RA and in those with depression [55]. Because the inclusion group differed from the exclusion group in terms of age and use of therapeutic agents, these results could lead to the selection bias. Although in the general population, but not in RA patients, meta-analyses and RCT studies have proven the effectiveness of dietary interventions [17, 18, 43], there remains the possibility of reverse causation as depression itself could promote unfavorable lifestyle choices such as unhealthy eating habits. We did not evaluate medical adherence although it was a confounding factor related to depression and anxiety status. In addition, there still could remain unadjusted confounding variables including exercise and smoking status, which could influence both the RA disease activity and mental health [53]. Finally, both the HADS score and FFQ are self-reported assessment tools and might not reflect the objective depression state or the actual food consumption.

## Conclusions

In conclusion, depression state showed a significant negative association with fish intake frequency and a positive one with disability in a RA population. Dietary intervention such as encouragement of fish consumption as well as the control of RA disease activity could be a potential clinical approach to improve the mental health of RA patients.

## Supporting information

S1 Fig Trends of anxiety score by intake frequency. The vertical axis represents HADS anxiety score and the horizontal axis represents intake frequency of each category. 1 out of 20 groups are negatively associated with HADS anxiety score; fish. P_trend_ values are calculated by a Jonckheere-Terpstra trend test. Abbreviations: *HADS* hospital anxiety and depression scale.
(TIF)

S1 Table Comparison of baseline demographic and clinical data for included and excluded population. Results of comparisons of characteristic between included and excluded patients. Data are presented as the mean (± standard deviation) or mean (interquartile range: IQR) for continuous variables, and as numbers (%) for categorial variables. * Steinbrocker’s classification. Abbreviations: *CRP* C-reactive protein, *RA* rheumatoid arthritis.
(DOCX)

S2 Table Multivariate analyses for independent factors associated with anxiety scores including factors of dietary habits. Results of multiple regression analysis with dietary habits and RA-related factors. Model was adjusted for RA duration, dietary habits (intake frequency of fish), RA therapeutics (use of prednisolone, biologics and methotrexate) and the patients’ status of disability (model 1) or DAS28-CRP remission (model 2) or both variables (model 3). Binary variables were constructed as follows: DAS28-CRP (0: < 2.6, 1: ≥2.6) and Patients without disability (HAQ-DI; 0: >0.5, 1: ≤ 0.5), fish (0: low frequency (≤ 2 times/weeks, 1: high frequency (≥3 times/week)). Abbreviations: *HADS* hospital anxiety and depression scale, *DAS28-CRP* 28-joint Disease Activity Score using C-reactive protein, *HAQ* health assessment questionnaire, *RA* rheumatoid arthritis.
(DOCX)

S1 Appendix The food frequency questionnaire of this study. (DOCX)

S2 Appendix The questionnaire of HADS (hospital anxiety and depression scale). (DOCX)

S1 Data (XLSX)
