# Supplementary figures and images for: Influence of dietary habits on depression among patients with rheumatoid arthritis: A cross-sectional study using KURAMA cohort database
Source: PLoS One. 2021 Aug 5;16(8):e0255526. doi: 10.1371/journal.pone.0255526 (PMC8341538; doi:10.1371/journal.pone.0255526)

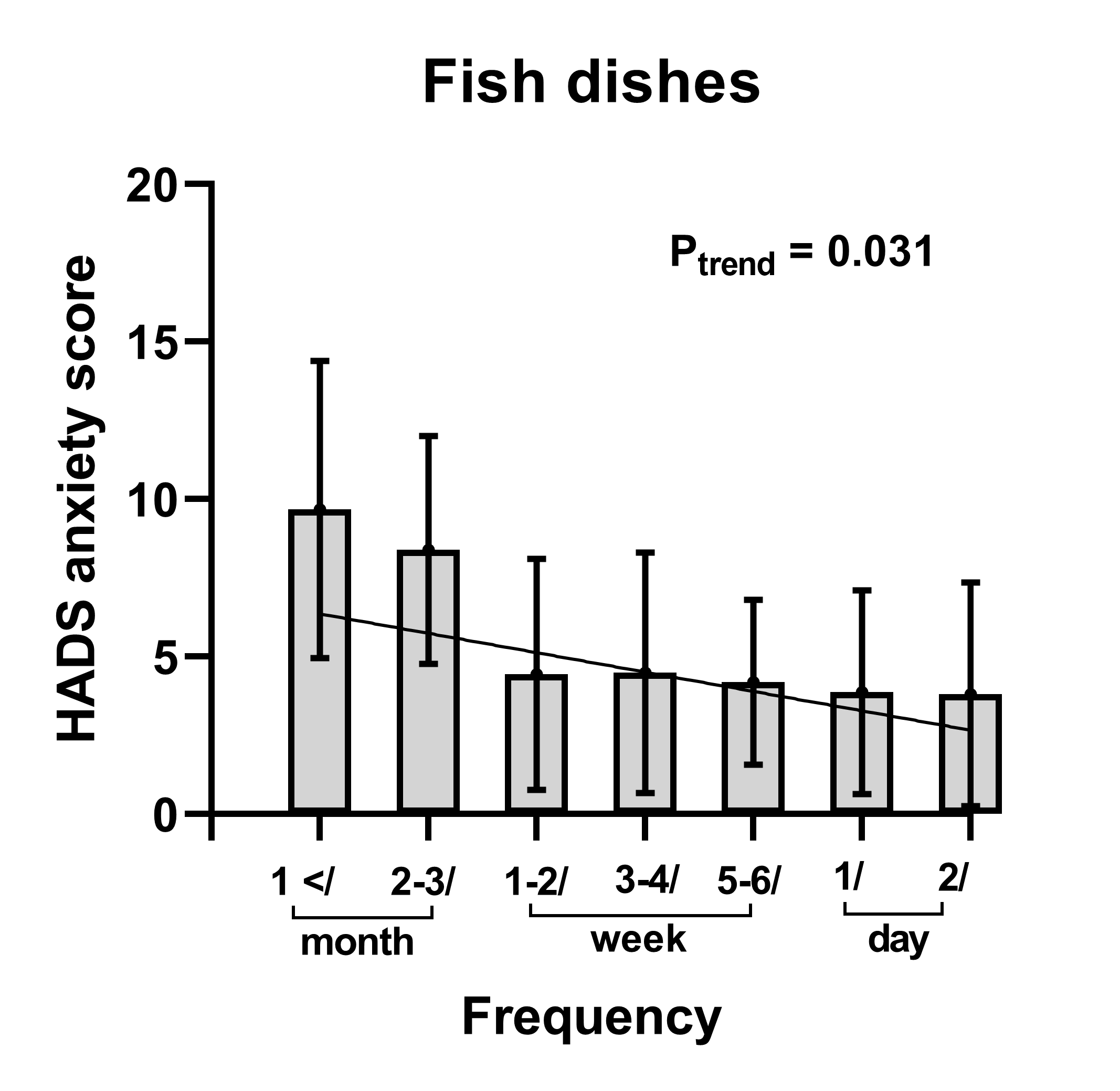

Supplement: S1 Fig — The vertical axis represents HADS anxiety score and the horizontal axis represents intake frequency of each category. 1 out of 20 groups are negatively associated with HADS anxiety score; fish. Ptrend values are calculated by a Jonckheere-Terpstra trend test. Abbreviations: HADS hospital anxiety and depression scale. (TIF) [file pone.0255526.s001.tif]
