# Supplementary material for: Influence of dietary habits on depression among patients with rheumatoid arthritis: A cross-sectional study using KURAMA cohort database
Source: PLoS One. 2021 Aug 5;16(8):e0255526. doi: 10.1371/journal.pone.0255526 (PMC8341538; doi:10.1371/journal.pone.0255526)
Supplement: S1 Table — Results of comparisons of characteristic between included and excluded patients. Data are presented as the mean (± standard deviation) or mean (interquartile range: IQR) for continuous variables, and as numbers (%) for categorial variables. * Steinbrocker’s classification. Abbreviations: CRP C-reactive protein, RA rheumatoid arthritis. (DOCX) [file pone.0255526.s002.docx]

**S1 Table. Comparison of baseline demographic and clinical data for included and excluded group.**

|  |  |  | Included patients | Excluded patients |  |
| --- | --- | --- | --- | --- | --- |
| Items | | | (n = 267) | (n = 98) | ***P* value *** |
| Age, years | | | 60.7 ± 12.8 | 65.5 ± 13.3 | 0.002 |
| Body mass index, kg/m2 | | | 21.9 ± 3.5 | 21.8 ± 4.1 | 0.72 |
| Laboratory data | | |  |  |  |
|  | Hemoglobin, g/dL | | 12.4 ± 1.4 | 12.5 ± 1.5 | 0.78 |
|  | Albumin, g/dL | | 3.91 ± 0.33 | 3.80 ± 0.53 | 0.024 |
|  | CRP, mg/dL | | 0.1 (0 - 0.2) | 0.1 (0 - 0.3) | 0.065 |
| RA-related parameters | | |  |  |  |
|  | Duration, years | | 13.4 ± 12.6 | 14.6 ± 12.5 | 0.41 |
|  | Stage* | | 2.73 ± 1.18 | 2.82 ± 1.15 | 0.52 |
|  |  | Stage I, *n* (%) | 55 (20.6) | 18 (18.4) | 0.77 |
|  |  | Stage I, II, *n* (%) | 121 (45.3) | 39 (39.8) | 0.40 |
|  |  | Stage I, II, III, *n* (%) | 164 (61.4) | 59 (60.2) | 0.90 |
| RA therapeutics | | |  |  |  |
|  | Methotrexate use, *n* (%) | | 198 (74.2) | 54 (55.1) | 0.008 |
|  | Prednisolone use, *n* (%) | | 75 (28.1) | 37 (37.8) | 0.10 |
|  | Biological agent use, *n* (%) | | 123 (46.1) | 63 (64.3) | 0.095 |

**S1 Table. Comparison of baseline demographic and clinical data for included and excluded group.**

Results of comparisons of characteristic between included and excluded participants. Data are presented as the mean (± standard deviation) or mean (interquartile range: IQR) for continuous variables, and as numbers (%) for categorial variables.

* Steinbrocker's classification

Abbreviations: *CRP* C-reactive protein, *RA* rheumatoid arthritis
