# Supplementary material for: Influence of dietary habits on depression among patients with rheumatoid arthritis: A cross-sectional study using KURAMA cohort database
Source: PLoS One. 2021 Aug 5;16(8):e0255526. doi: 10.1371/journal.pone.0255526 (PMC8341538; doi:10.1371/journal.pone.0255526)
Supplement: S2 Table — Results of multiple regression analysis with dietary habits and RA-related factors. Model was adjusted for RA duration, dietary habits (intake frequency of fish), RA therapeutics (use of prednisolone, biologics and methotrexate) and the patients’ status of disability (model 1) or DAS28-CRP remission (model 2) or both variables (model 3). Binary variables were constructed as follows: DAS28-CRP (0: < 2.6, 1: ≥2.6) and Patients without disability (HAQ-DI; 0: >0.5, 1: ≤ 0.5), fish (0: low frequency (≤ 2 times/weeks, 1: high frequency (≥3 times/week)). Abbreviations: HADS hospital anxiety and depression scale, DAS28-CRP 28-joint Disease Activity Score using C-reactive protein, HAQ health assessment questionnaire, RA rheumatoid arthritis. (DOCX) [file pone.0255526.s003.docx]

**S2 Table.　Multiple regression analysis for independent factors associated with HADS anxiety scores**

|  |  |  | **model 1** | | **model 2** | | **model 3** | |
| --- | --- | --- | --- | --- | --- | --- | --- | --- |
| **Dependent variables** | **Independent variables** |  | **Estimates** | ***p-*value** | **Estimates** | ***p-*value** | **Estimates** | ***p-*value** |
| Anxiety score (HADS) | Dietary habits | Fish dishes | -0.39 | 0.11 | -0.37 | 0.12 | -0.39 | 0.10 |
|  | Current disease activity | DAS28-CRP remission |  |  | -0.24 | 0.44 | 0.41 | 0.21 |
|  | Physical function | Patients without disability | -1.09 | < 0.0001 |  |  | -1.22 | < 0.0001 |
|  |  | Prednisolone (+) | -0.14 | 0.59 | -0.11 | 0.67 | -0.10 | 0.70 |
|  |  | Biological agents (+) | -0.14 | 0.56 | -0.10 | 0.68 | -0.19 | 0.44 |
|  |  | MTX (+) | -0.34 | 0.20 | -0.34 | 0.15 | -0.34 | 0.21 |
|  |  | duration (10 years) | -0.24 | 0.22 | 0.051 | 0.78 | -0.23 | 0.23 |

**S2 Table. Multivariate analyses for independent factors associated with anxiety scores including factors of dietary habits**

Results of multiple regression analysis with dietary habits and RA-related factors. Model was adjusted for RA duration, dietary habits (intake frequency of fish), RA therapeutics (use of prednisolone, biologics and methotrexate) and the patients’ status of disability (model 1) or DAS28-CRP remission (model 2) or both variables (model 3).

Binary variables were constructed as follows: DAS28-CRP (0: 2.6 ≤, 1: < 2.6) and Patients without disability (HAQ; 0: 0.5 <, 1: ≤ 0.5), fish (0: low frequency (≤ 2 times/weeks, 1: high frequency (≥3 times/week)).

Abbreviations: *HADS* hospital anxiety and depression scale, *DAS28-CRP* 28-joint Disease Activity Score using C-reactive protein, *HAQ* health assessment questionnaire, *RA* rheumatoid arthritis
