# Supplementary material for: Influence of dietary habits on depression among patients with rheumatoid arthritis: A cross-sectional study using KURAMA cohort database
Source: PLoS One. 2021 Aug 5;16(8):e0255526. doi: 10.1371/journal.pone.0255526 (PMC8341538; doi:10.1371/journal.pone.0255526)
Supplement: S1 Appendix — (DOCX) [file pone.0255526.s004.docx]

**S1 Appendix.　The food frequency questionnaire of this study**

**How many times did you eat these foods during the last 1 year?**

**Read each item and tick only one box which comes closest to how often you eat in the last 1 year.**

| **Foods** | **Intake frequency** | | | | | | | |
| --- | --- | --- | --- | --- | --- | --- | --- | --- |
|  | Less than 1 time/month | 1-3 times/month | 1-2 times/week | 3-4 times/week | 5-6 times/week | Once daily | Twice daily | Three times daily |
| 1. Staple food (bread, noodles, or rice) for breakfast | □ | □ | □ | □ | □ | □ | □ | □ |
| 2. Staple food for lunch | □ | □ | □ | □ | □ | □ | □ | □ |
| 3. Staple food for dinner | □ | □ | □ | □ | □ | □ | □ | □ |
| 4. Meat | □ | □ | □ | □ | □ | □ | □ | □ |
| 5. Fish | □ | □ | □ | □ | □ | □ | □ | □ |
| 6. *Tofu* (soybean curd) | □ | □ | □ | □ | □ | □ | □ | □ |
| 7. Eggs | □ | □ | □ | □ | □ | □ | □ | □ |
| 8. Milk | □ | □ | □ | □ | □ | □ | □ | □ |
| 9. Vegetables | □ | □ | □ | □ | □ | □ | □ | □ |
| 10. Fruits | □ | □ | □ | □ | □ | □ | □ | □ |
| 11. Deep-fried food (including *Tempura*) | □ | □ | □ | □ | □ | □ | □ | □ |
| 12. Cakes or Japanese sweets | □ | □ | □ | □ | □ | □ | □ | □ |
| 13. Juice or isotonic drinks | □ | □ | □ | □ | □ | □ | □ | □ |
| 14. Junk food | □ | □ | □ | □ | □ | □ | □ | □ |
| 15. Sweets like candies and chocolates | □ | □ | □ | □ | □ | □ | □ | □ |
| 16. Frozen foods | □ | □ | □ | □ | □ | □ | □ | □ |
| 17. Pickles | □ | □ | □ | □ | □ | □ | □ | □ |
| 18. Processed food (ham, sausage or *kamaboko* (boiled fish paste)) | □ | □ | □ | □ | □ | □ | □ | □ |
| 19. *miso* soup (fermented soybean paste) | □ | □ | □ | □ | □ | □ | □ | □ |
| 20. Alcohol | □ | □ | □ | □ | □ | □ | □ | □ |
