# Supplementary material for: Influence of dietary habits on depression among patients with rheumatoid arthritis: A cross-sectional study using KURAMA cohort database
Source: PLoS One. 2021 Aug 5;16(8):e0255526. doi: 10.1371/journal.pone.0255526 (PMC8341538; doi:10.1371/journal.pone.0255526)
Supplement: S2 Appendix — (DOCX) [file pone.0255526.s005.docx]

**S2 Appendix.　The questionnaire of HADS (Hospital Anxiety and Depression Scale)**

**Read each item and tick only one box which comes closest to how you have been feeling in the past week.**

| **I feel tense or 'wound up':** | | | |  | **Check box** | | **Depression** | **Anxiety** |
| --- | --- | --- | --- | --- | --- | --- | --- | --- |
|  | Most of the time | |  |  | | □ |  | 3 |
|  | A lot of the time | |  |  | | □ |  | 2 |
|  | From time to time, occasionally | | |  | | □ |  | 1 |
|  | Not at all |  |  |  | | □ |  | 0 |
|  |  |  |  |  | |  |  |  |
| **I still enjoy the things I used to enjoy:** | | | | | |  |  |  |
|  | Definitely as much | |  |  | | □ | 0 |  |
|  | Not quite so much | |  |  | | □ | 1 |  |
|  | Only a little | |  |  | | □ | 2 |  |
|  | Hardly at all | |  |  | | □ | 3 |  |
|  |  |  |  |  | |  |  |  |
| **I get a sort of frightened feeling as if something awful is about to happen:** | | | | | | | | |
|  | Very definitely and quite badly | | |  | | □ |  | 3 |
|  | Yes, but not too badly | | |  | | □ |  | 2 |
|  | A little, but it doesn't worry me | | |  | | □ |  | 1 |
|  | Not at all |  |  |  | | □ |  | 0 |
|  |  |  |  |  | |  |  |  |
| **I can laugh and see the funny side of things:** | | | | | | |  |  |
|  | As much as I always could | | |  | | □ | 0 |  |
|  | Not quite so much now | | |  | | □ | 1 |  |
|  | Definitely not so much now | | |  | | □ | 2 |  |
|  | Not at all |  |  |  | | □ | 3 |  |
|  |  |  |  |  | |  |  |  |
| **Worrying thoughts go through my mind:** | | | | | |  |  |  |
|  | A great deal of the time | | |  | | □ |  | 3 |
|  | A lot of the time | |  |  | | □ |  | 2 |
|  | From time to time, but not too often | | | | | □ |  | 1 |
|  | Only occasionally | |  |  | | □ |  | 0 |
|  |  |  |  |  | |  |  |  |
| **I feel cheerful:** | |  |  |  | |  |  |  |
|  | Not at all |  |  |  | | □ | 0 |  |
|  | Not often |  |  |  | | □ | 1 |  |
|  | Sometimes | |  |  | | □ | 2 |  |
|  | Most of the time | |  |  | | □ | 3 |  |
|  |  |  |  |  | |  |  |  |
| **I can sit at ease and feel relaxed:** | | | |  | |  |  |  |
|  | Definitely |  |  |  | | □ |  | 3 |
|  | Usually |  |  |  | | □ |  | 2 |
|  | Not Often |  |  |  | | □ |  | 1 |
|  | Not at all |  |  |  | | □ |  | 0 |
|  |  |  |  |  | |  |  |  |
| **I feel as if I am slowed down:** | | | |  | |  |  |  |
|  | Nearly all the time | |  |  | | □ | 0 |  |
|  | Very often |  |  |  | | □ | 1 |  |
|  | Sometimes | |  |  | | □ | 2 |  |
|  | Not at all |  |  |  | | □ | 3 |  |
|  |  |  |  |  | |  |  |  |
| **I get a sort of frightened feeling like 'butterflies' in the stomach:** | | | | | | | |  |
|  | Not at all |  |  |  | | □ |  | 3 |
|  | Occasionally | |  |  | | □ |  | 2 |
|  | Quite Often | |  |  | | □ |  | 1 |
|  | Very Often | |  |  | | □ |  | 0 |
|  |  |  |  |  | |  |  |  |
| **I have lost interest in my appearance:** | | | | | |  |  |  |
|  | Definitely |  |  |  | | □ | 0 |  |
|  | I don't take as much care as I should | | | | | □ | 1 |  |
|  | I may not take quite as much care | | | | | □ | 2 |  |
|  | I take just as much care as ever | | |  | | □ | 3 |  |
|  |  |  |  |  | |  |  |  |
| **I feel restless as I have to be on the move:** | | | | | |  |  |  |
|  | Very much indeed | |  |  | | □ |  | 3 |
|  | Quite a lot | |  |  | | □ |  | 2 |
|  | Not very much | |  |  | | □ |  | 1 |
|  | Not at all |  |  |  | | □ |  | 0 |
|  |  |  |  |  | |  |  |  |
| **I look forward with enjoyment to things:** | | | | | |  |  |  |
|  | As much as I ever did | | |  | | □ | 0 |  |
|  | Rather less than I used to | | |  | | □ | 1 |  |
|  | Definitely less than I used to | | |  | | □ | 2 |  |
|  | Hardly at all | |  |  | | □ | 3 |  |
|  |  |  |  |  | |  |  |  |
| **I get sudden feelings of panic:** | | | |  | |  |  |  |
|  | Very often indeed | |  |  | | □ |  | 3 |
|  | Quite often | |  |  | | □ |  | 2 |
|  | Not very often | |  |  | | □ |  | 1 |
|  | Not at all |  |  |  | | □ |  | 0 |
|  |  |  |  |  | |  |  |  |
| **I can enjoy a good book or radio or TV program:** | | | | | | |  |  |
|  | Often |  |  |  | | □ | 0 |  |
|  | Sometimes | |  |  | | □ | 1 |  |
|  | Not often |  |  |  | | □ | 2 |  |
|  | Very seldom | |  |  | | □ | 3 |  |

**Scoring:**

　Depression ____ /Anxiety____ (Total score of each item)

　0-7 … No

　8-10… Possible

　11-21… Probable
